# No Link Between Speech-in-Noise Perception and Auditory Sensory Memory – Evidence From a Large Cohort of Older and Younger Listeners

**DOI:** 10.1177/23312165231190688

**Published:** 2023-10-13

**Authors:** Roberta Bianco, Maria Chait

**Affiliations:** 1 Ear Institute, 4919 University College London, London, UK; 2 Neuroscience of Perception and Action Lab, 378790 Italian Institute of Technology (IIT), Rome, Italy

**Keywords:** CRM, temporal regularity, short-term memory, ageing, online testing

## Abstract

A growing literature is demonstrating a link between working memory (WM) and speech-in-noise (SiN) perception. However, the nature of this correlation and which components of WM might underlie it, are being debated. We investigated how SiN reception links with auditory sensory memory (aSM) – the low-level processes that support the short-term maintenance of temporally unfolding sounds. A large sample of old (*N* = 199, 60–79 yo) and young (*N* = 149, 20–35 yo) participants was recruited online and performed a coordinate response measure-based speech-in-babble task that taps listeners’ ability to track a speech target in background noise. We used two tasks to investigate implicit and explicit aSM. Both were based on tone patterns overlapping in processing time scales with speech (presentation rate of tones 20 Hz; of patterns 2 Hz). We hypothesised that a link between SiN and aSM may be particularly apparent in older listeners due to age-related reduction in both SiN reception and aSM. We confirmed impaired SiN reception in the older cohort and demonstrated reduced aSM performance in those listeners. However, SiN and aSM did not share variability. Across the two age groups, SiN performance was predicted by a binaural processing test and age. The results suggest that previously observed links between WM and SiN may relate to the executive components and other cognitive demands of the used tasks. This finding helps to constrain the search for the perceptual and cognitive factors that explain individual variability in SiN performance.

## Introduction

Speech understanding in noisy environments (e.g., following an announcement at a train station or a friend's voice in the pub) depends not only on hearing acuity but also on a host of cognitive skills including attention, memory and executive function that support listeners’ ability to segregate, track and attend to a ‘target’ signal among interference (Heinrich et al., 2015; Holmes & Griffiths, 2019; Holmes et al., 2021; Kim et al., 2020; Lad et al., 2020; Moore et al., 2014; Roberts & Allen, 2016). Identifying the cognitive factors that affect listening outcomes in crowded scenes is a critical prerequisite for interpreting individual variability and understanding the challenges listeners with different cognitive profiles might face during listening. The latter is particularly pertinent for the characterisation of listening deficits in ageing individuals who, in addition to impaired peripheral auditory processing, also exhibit a decline in various cognitive abilities that might affect listening (Cowan et al., 2006; Dryden et al., 2017; Füllgrabe et al., 2015; Garami et al., 2020; Glisky, 2007; Greene & Naveh-Benjamin, 2020; Herrmann & Butler, 2021; Naveh-Benjamin & Kilb, 2014; Panza et al., 2015; Salthouse, 2004; Schneider & Pichora-Fuller, 2000; Wayne & Johnsrude, 2015).

In recent years, working memory (WM) has attracted substantial interest as a potentially important predictor of speech processing performance in noisy conditions (Füllgrabe & Rosen, 2016; Pichora-Fuller & Singh, 2006). WM refers to the cognitive processes that underpin the temporary storage and manipulation of information in a heightened state of availability (Baddeley, 2003; Christophel et al., 2017; Cowan, 2017; Ma et al., 2014). It can thus be conceptualised as an interplay of multiple functions including: (i) short-term storage of low-level sensory information (sensory memory), (ii) active transformation and active maintenance of this information and (iii) executive processes that support the interface between remembered information and memory-guided behaviours (Baddeley & Hitch, 1974; Daneman & Carpenter, 1980). Commonly used tasks tap differentially into these distinct aspects. For example, the forward digit span task predominantly draws on the short-term memory storage component (Karpicke & Pisoni, 2004; Richardson, 2007; participants are asked to repeat serially presented digits in order) whilst tasks such as the reading span task load executive processes (Akeroyd, 2008; Bopp & Verhaeghen, 2005; Daneman & Merikle, 1996; Gordon-Salant & Cole, 2016; Smith & Pichora-Fuller, 2015; participants are asked to read a series of unconnected sentences aloud and to remember the final word of each sentence). There is an extensive, and growing, literature about the potential role of WM in speech-in-noise (SiN) reception. Correlations between WM ability and SiN intelligibility performance have been demonstrated in multiple studies, but the interpretation of these findings remains debated. In particular, it is not clear whether observed effects indicate an interaction at early (sensory memory), or relatively late stages of speech understanding and whether they are general or specific to certain populations and/or experimental manipulations (Akeroyd, 2008; Dryden et al., 2017; Humes, 2013; Wayne & Johnsrude, 2015).

Across the literature, correlations between SiN and WM performance appear to be more pronounced in older, and/or hearing-impaired individuals than in younger, normal-hearing listeners (Akeroyd, 2008; Füllgrabe et al., 2015; Kim et al., 2020; Rönnberg et al., 2016; Rudner et al., 2011). This has been interpreted to suggest that WM does not play an obligatory role in speech processing in all listeners (Füllgrabe & Rosen, 2016). Rather, the observed correlations may indicate increased compensatory reliance on executive control mechanisms and reallocable processing resources to make up for degraded sensory encoding of the speech signal (Baldwin & Ash, 2011; Bosen & Barry, 2020; Füllgrabe, 2013; Füllgrabe & Rosen, 2016; Pichora-Fuller et al., 1995).

However, effects independent of listeners’ age and hearing sensitivity have also been reported (e.g., Gordon-Salant & Cole, 2016; Lad et al., 2020; Millman & Mattys, 2017). Using a non-word repetition task, Millman & Mattys (2017) demonstrated that only the low-level phonological, but not the executive component of WM was related to individual differences in SiN perception in listeners with normal hearing (31–67 years old). Because of the non-linguistic nature of the task, the phonological component likely reflects the temporary retention of sensory information. This suggests that listeners’ ability to identify speech in fluctuating backgrounds may specifically draw on short-term memory capacity. Similarly, Lad et al. (2020) reported that the SiN performance of normal-hearing participants (age range 18–53) was predicted by a non-speech-based WM task designed to tap into short-term auditory storage (participants actively adjusted the frequency of a pure tone to match a previously presented token).

The consolidation of the various findings is complicated by the multi-level nature of WM and the different tasks used to quantify it. To advance our understanding of the link between auditory memory and speech reception, it is critical to develop finer-grained measures of the various components hypothesised to play a role in WM, including executive and auditory sensory memory (aSM) processes.

Here, we focus on the association between SiN perception and aSM – the time- and capacity-limited processes responsible for temporarily retaining sound information in memory (Atkinson & Shiffrin, 1968; Cowan, 1984; Näätänen & Winkler, 1999). The hypothesis of this link has been proposed in the recent literature (Herrmann et al., 2022; Holmes & Griffiths, 2019) but not explicitly tested.

Because of the intrinsic temporal nature of sound, most listening tasks, including speech perception, require linking sequentially presented sensory information to form a coherent representation of an unfolding auditory object (Rimmele et al., 2015; Winkler et al., 2009). Evidence of robust implicit memory for arbitrary, complex sounds, such as noise (Agus et al., 2010; Kaernbach, 2004; McDermott et al., 2011), tone clouds (Agus & Pressnitzer, 2021; Kumar et al., 2016), or regularly repeating patterns in rapidly unfolding tone sequences (Barascud et al., 2016; Bianco et al., 2020) indicates that aSM plays a fundamental and automatic role in auditory processing. In turn, deficits in aSM may lead to impaired encoding of unfolding sounds, such as detailed acoustic information in the speech signal, potentially leading to difficulties in noisy environments (Fogerty et al., 2016; Pisoni, 1975).

According to established models of aSM (Atkinson & Shiffrin, 1968; Cowan, 2008; see also Harrison et al., 2020), the processing of auditory information involves a multi-stage system. Initially, information is automatically encoded and stored in an auditory-specific memory buffer, which exhibits high fidelity in preserving the sensory details of the stimuli. Subsequently, information is transferred to a second short-term store, thought to involve more active cognitive processes. Factors such as individual differences in cognitive abilities, deliberate encoding strategies employed by listeners, their domain expertise, and attentional resources can influence the efficiency and accuracy of this transfer (Talamini et al., 2017).

**Figure 1.**
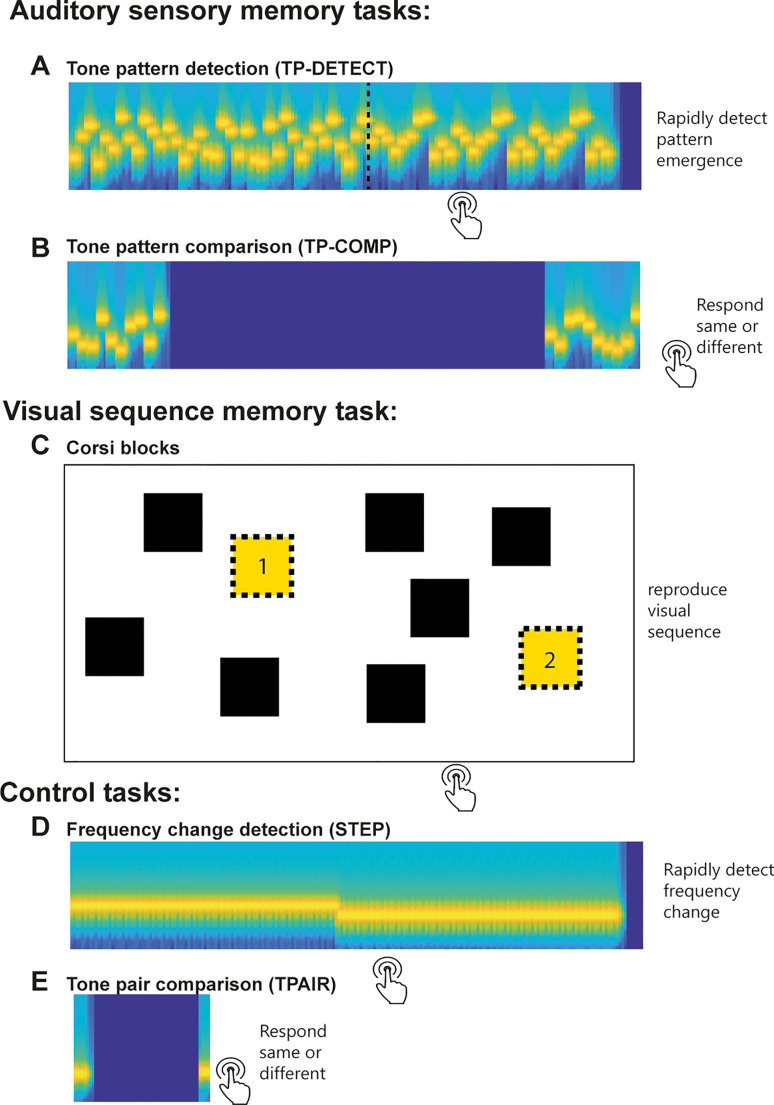
A Schematic Representation of the Main Tasks Used. Auditory sensory memory tasks included (A) Tone Pattern detection (TP-DETECT) and (B) Tone Pattern Comparison (TP-COMP) to probe automatic and active auditory sensory memory, respectively. In TP-DETECT participants had to respond, as quickly as possible, upon hearing a repeating 10 tone pattern emerging from the random sequence. TP-COMP required participants to compare two 10-tone sequences presented sequentially 2 s apart (same/different paradigm). (C) To measure visual sequence memory, we used the Corsi blocks task. Participants were required to remember and reproduce the temporal order of spatial sequences. Two control tasks included: (D) Frequency change detection (STEP) and tone pair comparison (TPAIR) to probe participants’ attention and engagement with low-demand tasks. In STEP, participants responded as quickly as possible to a change in frequency within a rapid tone-pip sequence. In TPAIR participants were required to compare the frequency of two 50 ms tone pips presented 500 ms apart.

We use a tone pattern detection task (TP-DETECT; Figure 1) to tap the **automatic aSM** components. This paradigm is extensively used to understand low-level aSM (Barascud et al., 2016; Bianco et al., 2020, 2023; Herrmann et al., 2021; Milne et al., 2021; Southwell et al., 2017). Short tones forming patterns are presented at time scales resembling those in speech (20-Hz tone presentation rate, 2-Hz pattern rate) (Rosen, 1992). The sequences are structured to contain a transition from a random pattern (RAN, tones are randomly arranged over time) to a regularly repeating pattern (REG, a pattern of 10 tones is repeated identically a few times). Sequences are novel on each trial, and participants are asked to respond to the emergence of the REG pattern by pressing a button. The sequences are rapid, precluding deliberate structure monitoring and allowing the repeating pattern to perceptually ‘pop-out’ automatically. Auditory pattern detection is hypothesised to be supported by an automatic process that continuously compares incoming sounds with information of the just past stimulus temporarily stored in memory. Indeed, brain-response signatures of TP-DETECT are observed in the brain of passive listeners distracted away from the sounds (Barascud et al., 2016; Herrmann et al., 2021, 2022; Herrmann & Johnsrude, 2018; Southwell & Chait, 2018). These implicit mnemonic processes are hypothesised to play a fundamental role in auditory scene analysis, including speech perception, by organising sensory input into coherent perceptual streams (Winkler et al., 2009).

We used an additional auditory memory test (‘Tone pattern compare’; TP-COMP; Figure 1) to tap the active aSM components. TP-COMP is based on very similar stimuli to TP-DETECT, but involves an active, explicit delayed match to sample memory task (deliberate memorisation and recall) (Albouy et al., 2013; Graves et al., 2019; Schulze et al., 2011). Participants were required to memorise a 500 ms tone pattern (10 random 50 ms tones), actively retain it for 2 s, and compare it to a subsequently presented probe pattern. The TP-COMP task is similar in its structure to the digit span task (Richardson, 2007; Woods et al., 2011) – the dominant measure of active auditory short-term memory – but is based on arbitrary, rapid tone patterns, that preclude rehearsal, allowing us to focus on low-level sensory representations. Therefore, relating performance on TP-DETECT and TP-COMP to SiN reception will help to determine whether SiN reception depends on aSM, and if so, whether the effects are underpinned by automatic storage per se or related to active, explicit memorisation.

Using an online experimental platform (Prolific and Gorilla), we recruited 148 young (aged 20 to 35) and 199 old (aged 60 to 79) participants who reported no known hearing problems. We measured their speech perception ability by means of a coordinate response measure (CRM) SiN task – an adaptive matrix-type speech-in-noise task (where listeners select two keywords out of a closed set of colours and digits) in the presence of a two male-speaker babble (as implemented in Bianco et al., 2021; de Kerangal et al., 2021; Messaoud-Galusi et al., 2011). The SiN task is particularly effective for the present investigation because it is characterised by low recruitment of semantic information and WM, instead relying on listeners’ ability to perceive fine-grained detail of the target speech – a process that has been previously hypothesised to draw on aSM (Pisoni, 1975).

Examining the relationship between SiN performance and TP-DETECT and TP-COMP in this large N group allows us to identify the presence of any shared variability between SiN perception and aSM. In particular, accumulating evidence suggests that ageing is associated with reduced aSM ability (Bellis et al., 2000; Bopp & Verhaeghen, 2005; Cooper et al., 2006; Dhrruvakumar & Yathiraj, 2021; Fogerty et al., 2016; Lu et al., 2005; Rimmele et al., 2012; Ruzzoli et al., 2012; Sur & Golob, 2020; Trainor & Trehub, 1989). Under the hypothesis that aSM supports SiN tracking, a link between SiN reception and aSM may be particularly salient in this population.

Several other control tasks (see Figure 1 and Methods) were included in the study. Notably, a visual-spatial sequence memory task – the Corsi blocks tapping task (Corsi, 1972; Kessels et al., 2000) was used as a measure of non-auditory memory. The task taps into short-term memory storage of sequentially presented visuo-spatial items which participants are instructed to reproduce in the right order. Performance on this task reduces with ageing (Beigneux et al., 2007; Fournet et al., 2012). We sought to replicate similar effects in our sample and to further explore whether visual sequence memory correlates with our measures of aSM.

As a standard practice, we also included a headphones screening test to ensure that online participants are using appropriate audio equipment (Milne et al., 2020). The test is based on a dichotic pitch percept, Huggins pitch (HP; Cramer & Huggins, 1958), that is audible when information in the Left and Right channels is delivered independently, and is therefore a convenient screen for headphones use. However, due to the test's intrinsic dependence on binaural processing, which exhibits an age-related decline, an increased incidence of failure was hypothesised and indeed observed in the older cohort. Considering these observations, we used failure on the headphones test as a (rough) measure of impaired binaural processing that was included as a predictor in the main regression analyses (see below).

## Methods

### Power Analysis

Based on a previous meta-analysis of the link between cognitive and SiN tasks (Akeroyd, 2008; Dryden et al., 2017), we expected weak to moderate effect sizes. A power analysis testing for linear multiple regression (in G*power software; alpha = .05, 1-beta = .95) based on f^2 ^= 0.15 and up to six predictors suggested that 146 subjects should be sufficient to reveal any effects.

### Participants

**Figure 2.**
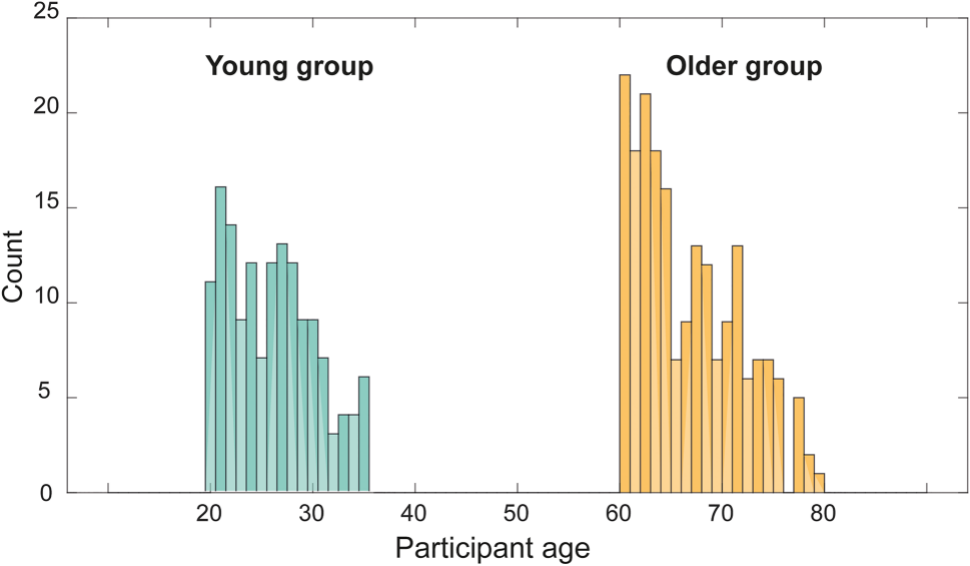
Participants' Age Distribution.

Two participant groups were tested (Figure 2). The older group comprised 199 participants (104 female; aged 60 to 79). Twenty-five of the participants were recruited from the ‘University of the 3^rd^ Age’ (U3A; https://www.u3a.org.uk/). The rest were recruited and compensated via the prolific crowdsourcing platform. Prolific has strict procedures for verifying participant age and identity. We therefore have high confidence in the demographic information. Additional inclusion criteria included being a native speaker of British English, general good health, and no known hearing problems. No other information on socioeconomic status and education was collected.

The younger group comprised 148 participants (96 female; aged 20 to 35; same inclusion criteria as above) who were also recruited and compensated via Prolific. Experimental procedures were approved by the research ethics committee of University College London and informed consent was obtained from each participant.

Though all participants self-identified as having no known hearing problems, the older cohort in particular is likely to have an increased prevalence of hearing loss. In a similar aged cohort tested in-lab about 50% of the participants had at least mild peripheral hearing loss (see de Kerangal et al., 2021, N = 41). We expect a comparable incidence in the online group.

### Procedure

**Figure 3.**
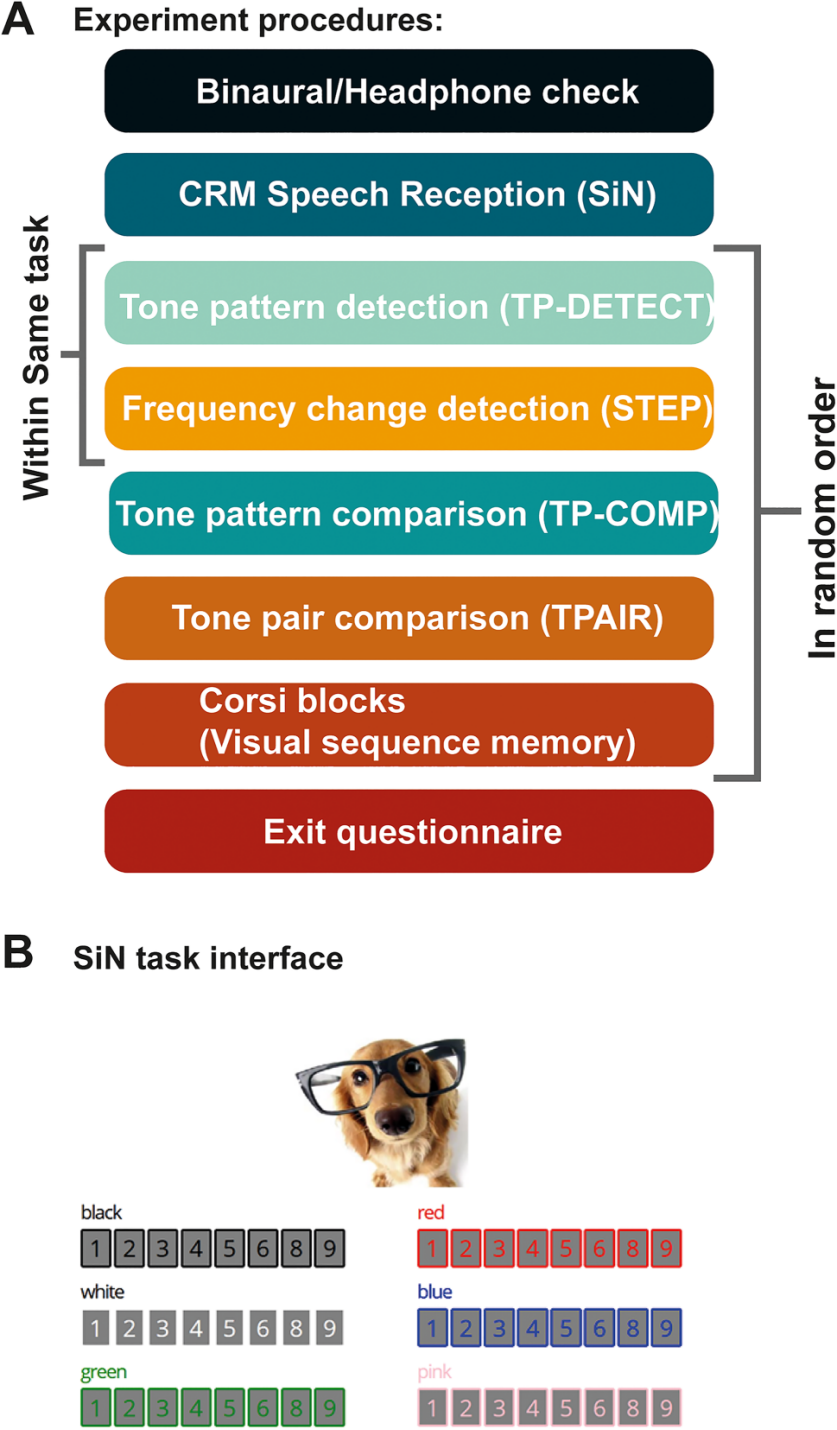
Experiment Procedure. (A) Experiment task order. After the binaural/headphones screen, participants performed the SIN task. This was followed by a battery of memory tests presented in random order across participants. (B) The participant interface from the SiN task. Following the presentation of the sentence ‘Show the dog where the (colour) (number) is’, participants mouse-clicked on the appropriate display element.

The experimental tasks were implemented in JavaScript. The Gorilla Experiment Builder platform (www.gorilla.sc) was used to host the experiment (Anwyl-Irvine et al., 2020). Tasks were performed in the order schematised in Figure 3A.

Participants first completed a headphones test (Milne et al., 2020), followed by the SiN task. The other tasks were completed subsequently, in random order (see details about each task below). To glean a better, qualitative understanding of the subjects’ auditory environment during testing, at the end of the experiment, participants completed a short questionnaire about their surroundings and equipment. We encouraged honest reports by stressing that ‘your answers will not affect your payment but will help us to get the best quality data’. Participants were asked whether they indeed used headphones (only 1 older and 1 younger participant admitted to not using headphones), whether they were disrupted at all during the experiment (yes/no), and how much background noise they experienced during the experiment (0 = silent and 10 = very noisy). Overall, the experiment took between 40–50 min to complete.

Our task implementation is openly available on the Gorilla platform (https://gorilla.sc/openmaterials/171870).

### Tasks

**Binaural/headphones screening task:** This test (Milne et al., 2020) is based on HP (Cramer & Huggins, 1958), an illusory pitch percept generated by presenting a white noise stimulus to one ear, and the same white noise – but with a phase shift of 180° over a narrow frequency band – to the other ear. HP is only detectable when L and R channels are presented separately to each ear and binaural hearing is not impaired. Each trial consisted of three sequentially presented intervals. Two of the intervals contained diotically presented white noise whilst the third interval contained the HP stimulus. Participants were asked to decide which of the three noises contained the tone by clicking on the appropriate button (1, 2 or 3). A total of six trials were presented. The main task was preceded by instructions and a demo (where the HP signal was replaced by a dioic pure tone). Participants were instructed to only progress to the main task if they could hear the demo tone. The task took approximately 3 min to complete.

Our usual practice with young participants has been not to allow those who failed the test to proceed with the study (e.g., Bianco et al., 2021). Here, because of uncertainty about the extent to which older people will exhibit difficulties with the binaural pitch stimulus, all participants were allowed to proceed irrespective of whether they passed or failed. We stressed that headphones use is critical for the study and subjects were repeatedly reminded that they must use headphones. The results of the screen (pass/fail, where the pass was assigned to those who correctly responded to 5/6 trials) were incorporated into later stages of data analysis (used as an independent predictor in the regression models). The results of the screen (see below) indicated a substantial difference between the older and young cohorts, presumably due to an age-related deficit in binaural processing. We therefore also used the test as a rough proxy for binaural processing ability and refer to it as ‘binaural/headphone’ test below.

**SiN task:** SiN reception was quantified with the speech recognition threshold (SRT) obtained for each participant using target sentences introduced by Messaoud-Galusi et al. (2011) – a modified version of the CRM corpus described by (Bolia et al., 2000). The same online implementation was previously used by Bianco et al. (2021). On each trial, participants heard a target sentence of the form ‘show the dog where the [colour] [number] is’. The number was a digit from 1 to 9, excluding the number 7 (due to its disyllabic phonetic structure, which would make it easier to identify). The colours were black, white, pink, blue, green, or red. Thus, there were a total of 48 combinations (six colours × eight numbers). Participants were instructed to press the correct combination of colour and number on a visual interface showing an image of a dog and a list of the digits in the different colours (Figure 3B).

The target sentences were spoken by a single female native speaker of Standard Southern British English that was presented simultaneously with a two male-speaker babble that the participants were instructed to ignore. Each talker in the babble was recorded reading two 5- to 6-sentence passages which were concatenated together once passages were edited to delete pauses of more than 100 ms. The two talkers were then digitally mixed at equal levels, with random sections of the appropriate duration from this 30-s long masker chosen for each trial.

The overall level of the mixture (target speaker + babble background) was kept fixed, with only the ratio between the target and masker changing on each trial. Mixtures were presented diotically (identically to the two ears). The signal-to-noise ratio (SNR) between the babble and the target speaker was initially set to 20 dB and was adjusted using a one-up one-down adaptive procedure, tracking the 50% correct threshold (Levitt, 1971). Initial steps were of 9 dB, decreasing by 2 dB following each reversal then stabilising at a final step size of 3 dB. The procedure terminated after seven reversals. The SRT was calculated as the mean of the SNRs in the last four reversals. Each participant performed the test in four consecutive runs of approximately 2 min each; the mean over the SNRs collected in the last three runs was used for the analyses. These parameters were chosen to allow for comparisons with our previous work (Bianco et al., 2021; de Kerangal et al., 2021). The task took approximately 10 min to complete.

**TP-DETECT:** A measure of implicit aSM (Barascud et al., 2016). Stimuli were sequences of 50-ms tone-pips (gated on and off with 5-ms raised cosine ramps) of different frequencies drawn from a pool of 20 log-spaced frequencies (range between 222 to 2000 Hz; 12% steps; loudness normalised based on iso226). RAN (‘random’) sequences consisted of 20 tone-pips arranged in random order, with the constraint that adjacent tones were not of the same frequency. Each frequency occurred equiprobably across the sequence duration. The RANREG (random-to-regular) condition contained a transition between a random (RAN) and a regularly repeating pattern (REG): sequences with initially randomly ordered tones changed into regularly repeating cycles of 10 frequencies randomly drawn from the pool (see Figure 1). The change (between 2000 and 2500 ms after sequence onset) was followed by 3 REG cycles (500 ms per cycle). RAN and RANREG conditions were generated anew for each trial and occurred equiprobably. Two additional conditions were included: sequences of tones of a fixed frequency (CONT), and sequences with a step change in frequency partway through the trial (STEP). The reaction times (RTs) to the step frequency change were analysed separately as a measure of task engagement (see below STEP task).

Participants were instructed to monitor for transitions from random to regular patterns (50% of trials) and press a keyboard button as soon as possible upon pattern detection. To acquaint participants with the task, a practice run of 26 trials was delivered (10 RAN, 10 RANREG, 4 STEP, 2 CONT). The main task consisted of two blocks of 3.5 min duration each. Each block contained 47 stimuli (20 RAN, 20 RANREG, 5 STEP, 2 CONT), with an inter-trial-interval of 1.3 s. Feedback on accuracy and speed was provided at the end of each trial. d’ was used as a measure of sensitivity to pattern presence. Responses to RANREG transition were considered hits; responses to RAN trials were considered false alarms. The task took approximately 18 min to complete.

**Frequency Change Detection (STEP) task:** RTs to STEP trials were computed as the time between the onset of the frequency change and the participant's button press. Only RTs of correct trials (hits) were analysed. This task was embedded in the TP-DETECT task to assess processing speed (Hultsch et al., 2002), vigilance and engagement.

**Tone Pattern Comparison (TP-COMP) task:** A measure of deliberate aSM (Albouy et al., 2013; Graves et al., 2019; Schulze et al., 2011). The stimuli contained two 500 ms tone-pip sequences separated by a 2000 ms silent gap (see Figure 1). The tone-pip sequences were comprised of ten 50 ms tone-pips drawn from the same pool described above (TP-DETECT task). Different patterns were drawn on each trial. The two sound sequences before and after the gap were matched on 50% of the trials (‘same’ trials) and differed in the other trials (‘different’ trials). The sequences in the ‘different’ trials were created by switching the positions of 5 of the 10 tones. The positions of the shuffled tones were randomly chosen on each trial, except for the first and last tones, to avoid the difference from becoming too obvious. The instructions were to listen carefully to the sound sequences and press one of two keyboard buttons to indicate whether the two-tone sequences were the same or different (‘S’ for same and ‘D’ for different). Participants then completed 32 trials. Feedback was provided. The correct response rate was used for the analyses. The task took approximately 5 min to complete.

**Tone Pair Comparison (TPAIR) task:** Stimuli were a pair of 50 ms tone pips, separated by a 500 ms silent gap (see Figure 1). Tone pip frequencies were drawn from the same frequency pool used for the TP-COMP and TP-DETECT tasks. The task required participants to determine whether the frequencies of tone pairs are the same or different. Only contiguous pairs of tones (12% frequency difference; two semi-tones) were used. Overall, each participant heard 39 pairs of tones (20 ‘same’; 19 ‘different’). Feedback was provided. The correct response rate was used for the analyses. The task took approximately 3 min to complete. TPAIR is an easy frequency discrimination task, which we expect most participants to do well in. Whilst it may identify participants with genuine frequency discrimination difficulties, the main challenge is associated with listening carefully to the briefly presented tone pips. We therefore interpret performance on this task as reflecting participant engagement and vigilance. See further discussion below.

**Corsi Blocks (visual-sequence memory) task:** A version of the classic Corsi blocks tapping task (Kessels et al., 2000) was implemented to assess visuospatial short-term memory. Nine identical black squares were presented on the screen. On each trial, following a fixation duration (500 ms) several blocks flashed (briefly changed colour from back to yellow; flash duration 500ms; inter-flash-interval 250 ms) in a sequence. Participants were required to reproduce the order by mouse-clicking on the correct blocks. The initial sequence length was two blocks. Correct responses result in a length increase and incorrect responses in a length decrease. Overall participants completed 20 trials. Two outcome measures were computed: (1) the maximum sequence length reached and (2) the mean sequence length completed across trials. We consider the latter to be a more sensitive measure of short-term memory and this value was therefore used for the analyses below (using the other measure yields an identical pattern of results). The task took approximately 5 min to complete.

To mitigate possible hearing loss, and further ensure that sounds are always presented at an easily audible level, a volume adjustment stage preceded each task: participants were played a few sounds, taken from the stimulus set of the upcoming task, and instructed to adjust the volume to as high a level as possible without it being uncomfortable.

Most participants completed all the tasks. Occasionally, due to technical issues, not all tasks were completed. All available data are used in the analyses below with missing data excluded pairwise.

### Analysis

The main analyses are based on generalised multiple (linear) regression models. Planned analyses tested separate models with SiN and TP-DETECT as dependent variables. In all cases, all the other available parameters (performance on all tasks as well as age) were included as predictors simultaneously (‘enter’ method). Following the observation that older listeners failed the binaural/headphones test more often than younger listeners, we also conducted an unplanned regression analysis to understand what factors might predict performance on the binaural/headphones test.

We additionally report (uncorrected) correlation analyses for confirmatory purposes, for example, to replicate known correlations between age and SiN performance and between age and Corsi blocks or to elaborate on results obtained in the regression analyses. Correlational analyses, where reported, adopted a conservative approach and used Spearman correlations. As expected from the literature (Dryden et al., 2017) in most cases correlation values are weak-moderate. Analysis was conducted in SPSS, MATLAB, and R.

## Results

### Older Participants Report Quieter Surroundings Than Younger Participants

The exit questionnaire asked participants about noise and disruption in their surroundings. The majority reported quiet, distraction-free settings. On average, older participants reported quieter environments (mean Older: 1.02 ± 1.6: Younger: 1.3 ± 1.6; independent samples Mann U Whitney test; U = 14973.5, *p* = .025).

### Older Participants Failed the Binaural/Headphones Screen More Often Than Younger Participants

The experiment began with the binaural/headphones test (Milne et al., 2020) based on HP (Akeroyd, 2008; Chait et al., 2006; Cramer & Huggins, 1958). Previous work (Akeroyd, 2008; Santurette & Dau, 2007, 2012) has shown that HP is the most easily perceivable binaural pitch, including in listeners with hearing impairment, which made it a particularly appealing stimulus for use as a headphones screen. The test is designed to distinguish between people listening over headphones, from those listening without headphones (e.g., over speakers), but users may also fail the test due to low-quality equipment, a noisy environment or if they have impaired binaural auditory processing. The latter is of particular concern when testing older participants. We therefore did not use the test to exclude participants from the study. Instead, outcomes were used as predictors in the subsequent analyses.

Eighty percent of the younger participants passed the binaural/headphones screen. In contrast, only 61% of the older participants passed the test. As can be seen from Table 1, performance on the binaural/headphones screen correlated with performance on the other tasks, but with some differences between the young and older groups. Linear regression was calculated to predict performance on the binaural/headphones test based on performance on SiN, TP-COMP, TPAIR, CORSI, TP-DETECT, and STEP as well as reported environmental noise and age. In the younger group, a significant regression equation was found, *F*(8, 129) = 5.56 *p* < .0001 with an R^2^ of 0.26. Only environmental noise (*p* < .0001; β = −0.29) and TPAIR (p = 0.039; β = 0.19) added significantly to the model. This indicates that noisier settings and poor performance on the attention check task predicted failure on the binaural/headphones check, consistent with the notion that failure on the binaural/headphones test among young listeners mostly reflects issues with equipment, environmental factors and engagement (see also Cooke & García Lecumberri, 2021).

**Table 1.** Correlations (Spearman Rho) Between Performance on the Binaural/Headphones Test and the Other Measures. Asterisks Indicate the Significance Level (*p < .050; **p < .010; ***p < .001). The Correlations in Bold Indicate Those Variables That Significantly Predicted Performance on the Binaural/Headphones Test in Linear Regression Models.

	SiN	TP-COMP	TP-DETECT	CORSI	TPAIR	STEP RT	Environmental noise	Age
Older	**Rho = ** **−0.375** *** ***p* < .0001**	Rho = 0.233 ** *p* = .001	Rho = 0.246 ** *p* = .001	Rho = 0.175 * *p* = .014	Rho = 0.230 ** *p* = .001	Rho = **−**0.257 *** *p* < .0001	Rho = **−**0.116 *p* = .110	Rho = **−**0.072 *p* = .312
Younger	Rho = **−**0.211 * *p* = .01	Rho = 0.180 * *p* = .029	Rho = 0.255 ** *p* = .002	Rho = 0.049 *p* = .557	**Rho = ** **0.235** ** ***p* = .004**	Rho = 0.025 *p* = .766	**Rho = ** **−0.307** *** ***p* < .0001**	Rho = 0.051 *p* = .538

In contrast, for older listeners (significant regression equation: *F*(8, 173) = 5.7; *p* < .0001; R^2 ^= 0.2) only SiN added significantly to the model (*p* < .0001; β = −0.285), indicating that poor speech reception performance predicted failure on the binaural/headphones test. This is despite the fact that stimuli in the SiN task were presented diotically (i.e., did not necessitate binaural processing per se).

These results, including the patterns of differences between groups, suggest that the poorer performance of the older listeners on the binaural/headphones test may reflect age-related auditory decline, which also affects binaural processing and hence sensitivity to the HP stimulus. The absence of correlation with age might be due to the narrow age range from which we sampled.

In the correlation analyses below, we explicitly control for performance on the binaural/headphones test. The reported linear regression analyses include performance on the binaural/headphones test as a predictor. We also excluded participants who reported Environmental noise >2. The mean for Environmental noise was 1.13 across groups (1.3 for younger; 1.02 for older). Therefore, a cut-off of 2 was selected. This allowed us to exclude 20% of the listeners who reported the loudest listening environments (remaining sample: *N* younger = 116; *N* older = 169; Total *N* = 285).

### SiN Performance Measured From Online Participants Was Overall Lower Than That Measured in Lab

We compared the SRT measured in the online group to data from similarly aged cohorts (older group: *N* = 83, 60–86 years old; younger group: *N* = 83; 20–38 years old) obtained in-lab (data from de Kerangal et al., 2021 and an additional unpublished set; see also Bianco et al., 2021). The in-lab test was conducted in a double-walled sound-proof booth (IAC, Winchester). The task, identical to the one used online, was implemented in MATLAB using a calibrated sound delivery system at a comfortable listening level (∼60–70 dB SPL), self-adjusted by each participant during the training block. Participants additionally underwent PTA testing which indicated a 50% prevalence of mild hearing loss in the older group (better ear thresholds > 25 dB HL for at least one frequency between 125 and 4000 Hz; see de Kerangal et al., 2021). It is plausible to assume a similar prevalence of hearing loss in our older online cohort, although the incidence may be even higher based on findings by Füllgrabe et al. (2015). It is worth noting that de Kerangal et al. excluded participants with more severe hearing loss, but there may be a proportion of such individuals in our online cohort despite self-declarations of ‘no hearing problems’. As we will see in the following analysis, there is evidence to support the conclusion that a proportion of the older participants, particularly those who failed the binaural/headphones test, have impaired hearing, which significantly contributes to their reduced SiN performance.

**Figure 4.**
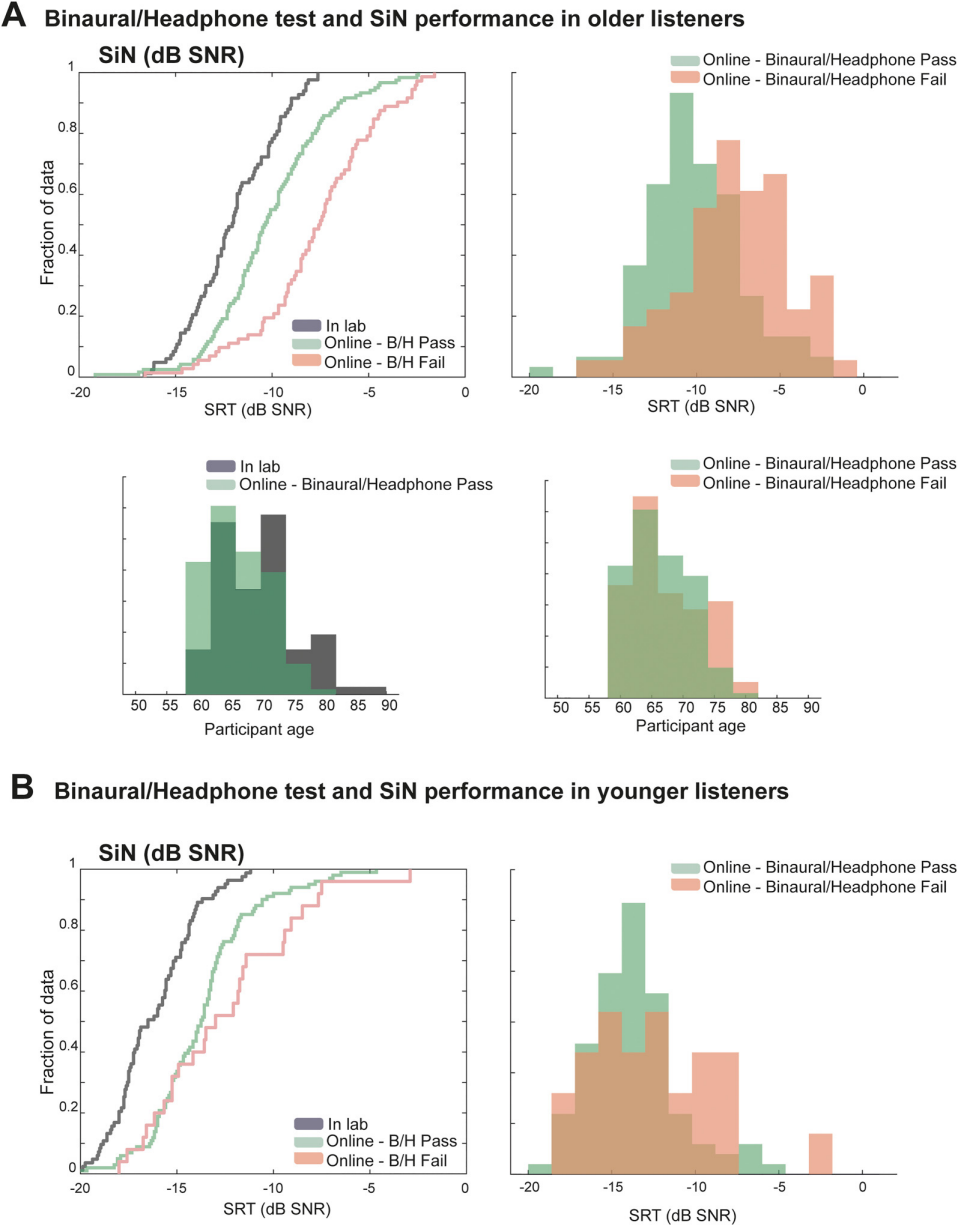
Binaural/Headphones Test and SiN Performance in Older and Younger Listeners**.** (A): Cumulative (left) and probability density (right) distribution of the in-lab and online SiN performance in older listeners. The online group is separated into those participants who passed or failed the binaural/headphones test. The bottom insets show the age histogram. (B): Cumulative (left) and probability density (right) distribution of the in-lab and online binaural/headphones pass and fail SiN performance in younger listeners.

Figure 4 shows the cumulative distribution functions and probability density functions of the SRT obtained from the SiN task in-lab and online groups. The online group is separated into those participants who passed or failed the binaural/headphones test. We used a two sample Kolmogorov–Smirnov (KS) test to ascertain the existence of a statistically significant difference between the (unknown) distributions of the in-lab and online groups.

For the older listeners: The KS test indicated a significant difference between the SRT distributions of those participants who passed versus failed the binaural/headphones test (D = 0.397, *p* < .0001). The maximal difference occurred at ∼10 dB, which was reached by 55% of the pass group and only by 19% of the ‘fail’ group. Though the two groups did not differ significantly by age, it does appear that there was a larger concentration of older participants (age > 75) in the ‘fail’ group, consistent with potential age-related hearing deficits that result in impaired binaural performance as well as SiN reception (though, notably, the SiN task was not binaural in nature).

We further observed a significant difference in SiN performance between the o*nline* ‘binaural/headphones pass’ group and the in-lab group (D = 0.344, *p* < .001), whereby the in-lab group exhibited SRT about 1.5 dB lower than the online group. This difference is not explainable by age (if anything participants in the *online* group are younger; see age histogram in bottom left), but might relate to environmental factors and motivation as speculated in Bianco et al. (2021).

In contrast, data from the younger listeners (Figure 4B) revealed a different pattern. The KS test indicated no significant difference between the SRT distributions of participants who passed versus failed the binaural/headphones test (*p* = .156). However, a significant difference was observed between the in-lab group and the *online ‘*binaural/headphones pass’ group (D = 0.439, *p* < .0001), with the online group exhibiting SRT approximately 3 dB higher than the in-lab group. As above, this difference is interpreted as a consequence of the lack of control over participants’ environment and motivation in the online setting, as previously discussed by Bianco et al. (2021).

Overall, these results suggest that while worse SiN performance can be expected in the online sample compared to the in-lab setting due to lower motivation and weaker environmental control, the greater reduction observed in the older online ‘binaural/headphones fail’ group may reflect the additional contribution of age-related auditory processing deficits which affect both binaural processing and SiN reception.

### As a Group, Older Participants Exhibited Worse SiN, Visual-Sequence Memory and Tone Pattern Detection Performance Than Young Listeners

**Figure 5.**
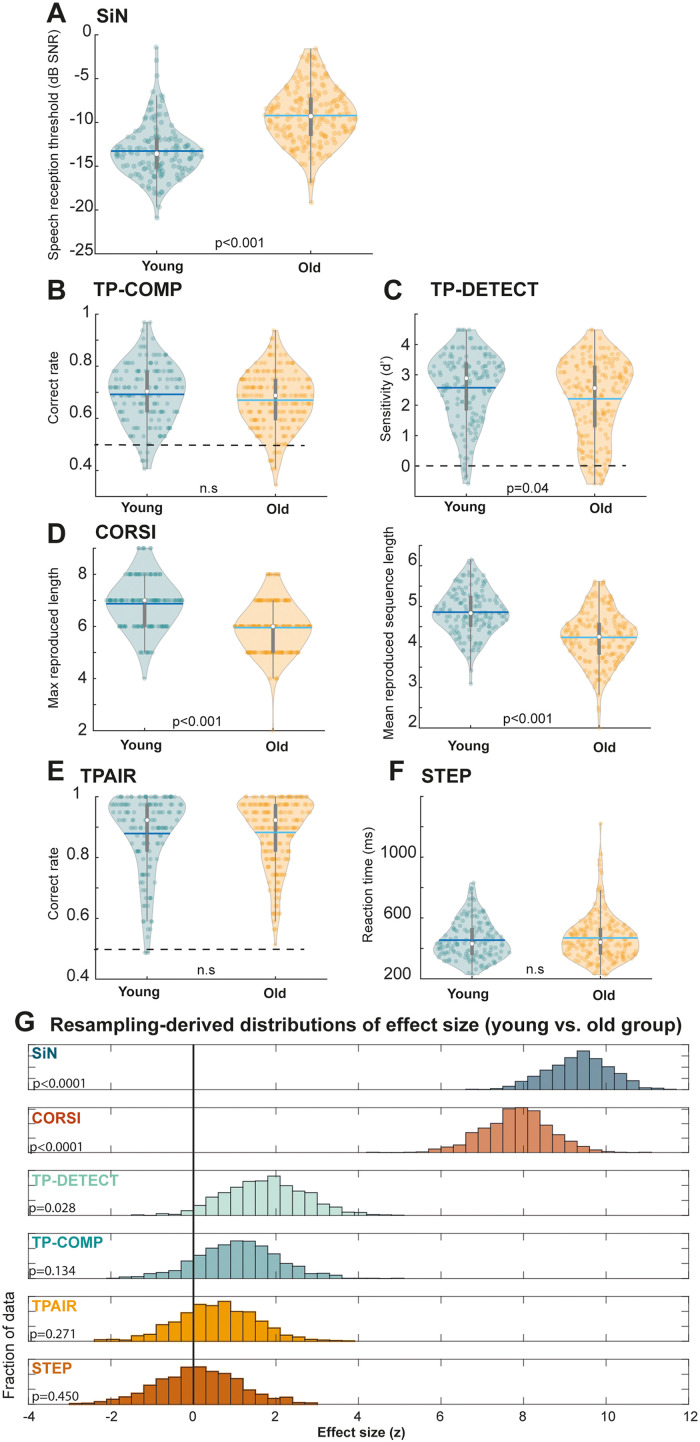
Distribution of Performance, Across the Various Tasks, in the Younger And Older Groups (*N* young = 116; *N* old = 169; total *N* = 285). Statistically significant differences between group means are indicated. Worse performance in the Older than in the Younger group was observed in the SiN task (A) and Corsi-blocks (D) (assessed as maximum reproduced length – left panel – and mean reproduced length across the 20 trials – right panel; the latter measure is used in the correlation/regression analyses below). We also observed a difference between groups in the TP-DETECT task (C). (G) Bootstrapped distributions of the effect sizes derived from the between-group comparisons in each task. The *p*-values quantify the proportion of iterations where the effect size was ≤ 0 (thick horizontal black line).

Figure 5 presents a comparison between the performance of the older and younger groups. All tasks were associated with substantial individual variability, with performance scores spanning the full range of possible performance levels. A one-tailed Mann–Whitney U test revealed that SiN (U = 3256, z = −9.332, *p* < .0001) and CORSI (U = 15159, z = 7.841, *p* < .0001) performance was significantly lower in the older group than in the younger group. This is consistent with previous reports (de Kerangal et al., 2021; Fournet et al., 2012). We additionally observed a difference in the TP-DETECT task (U = 10995, z = 1.7, *p* = .04), revealing lower sensitivity to transitions between random and regularly repeating tone patterns in the older group, in line with previous hypotheses (Herrmann et al., 2022). Performance on the other tasks did not differ between age groups (other *p*-values > .3).

We also analysed the effect sizes directly by bootstrap resampling. On each iteration (*N* = 1000) a group of Younger and Older participants were selected (with replacement; Ns equal to the total group sizes) and an effect size was derived from the Wilcoxon rank sum test comparing the two groups. Figure 5G presents the derived distributions of effect sizes for each task. This analysis demonstrates that the SiN and Corsi blocks tasks yielded the largest differences between younger and older listeners. Whilst substantially smaller, the distribution associated with TP-DETECT is significantly different from 0, suggesting a robust difference in means between the young and older groups. There was also a significant difference between the effect size distribution associated with TP-DETECT versus TP-COMP (two sample KS test: *p* < .0001, D = 0.258; Mann–Whitney U: z = 13.97, *p* < .0001) consistent with the former providing a more reliable measure of age-related decline in aSM.

### Within the Older Group, age Correlated With SiN and Visual-Sequence Memory Performance

To first confirm expectations based on previous literature, we tested for known links between ageing and SiN*.* Focusing on the older group, partial correlation analyses between age and task performance (controlling for performance on the binaural/headphones test) demonstrated significant, albeit weak, correlations only with SiN (r = 0.242; *p* = .002) and Corsi blocks (r = −0.183; *p* = .021) performance, confirming that both tasks are sensitive to age-related decline even when focusing on the 60–79 years range. SRT increased, and visual-spatial sequence memory performance decreased with increasing age, though the relatively skewed age distribution here might have limited the effect sizes observed.

### SiN Performance Did Not Correlate With Auditory Sensory Memory or Visual-Sequence Memory Performance

Critically, across the full sample, there was no significant correlation between SiN reception and aSM or visual-sequence memory performance. A regression analysis, within the full cohort of older and younger participants, predicting SiN performance with the other tasks (TP-DETECT, TP-COMP, CORSI, STEP, TPAIR, binaural/headphones task) and age as predictors were conducted. This analysis, *F*(7, 267) = 24.22, *p* < .001; 37% variance explained, indicated that only age (standardised β = 0.464; *p* < .001; likely reflecting the difference between the young and older groups since age was not continuously sampled) and binaural/headphones test performance (standardised β = −0.210; *p* < .001) added significantly to the model.

In the older group, a linear regression analysis with SiN as the dependent variable and the other tasks (TP-DETECT, TP-COMP, CORSI, STEP, TPAIR, binaural/headphones task) and age as predictors; *F*(7, 153) = 5.28, *p* < .001; 15.8% variance explained; confirmed that only age (standardised β = 0.196; *p* = .011) and binaural/headphones test performance (standardised β = −0.299; *p* < .001) added significantly to the model. Namely, performance on the SiN task was only predictable from binaural processing ability (as indirectly quantified with the binaural/headphones test) and participant's age. Consistently, a partial correlation analysis between the SiN task and the other factors (controlling for performance on the binaural/headphones test) demonstrated significant (but weak) correlations only with age (r = 0.242; *p* = .002).

A multiple regression analysis in the younger cohort yielded a non-significant model (*p* = .14), suggesting no effect of the factors considered in the present study on SiN reception among younger (20–35 years old) listeners.

### Auditory Pattern Detection Performance Is Linked With Both Visual-Sequence and Explicit Auditory Memory Tasks

Finally, we focus on the performance of the memory tasks. Specifically, we tested whether performance on the auditory pattern detection task (TP-DETECT), our key measure of aSM, shares variance with the other cognitive tasks.

**Figure 6.**
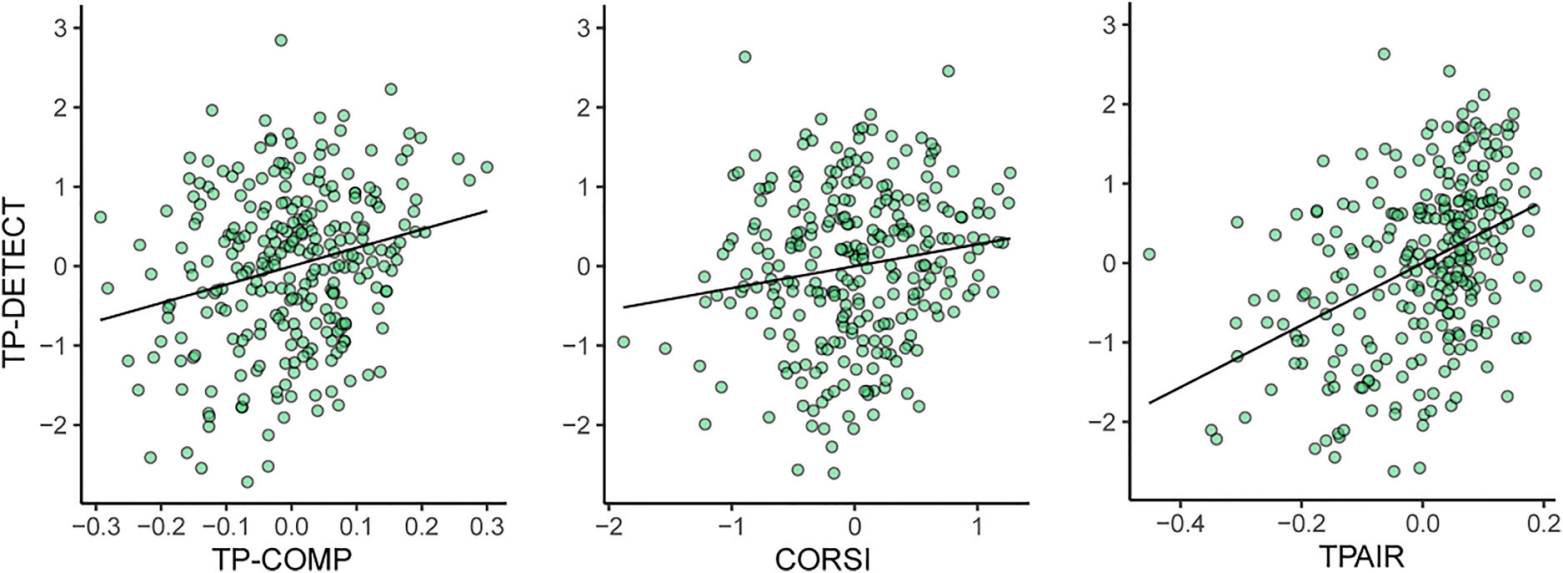
Partial Regression Plots Demonstrating the Link Between TP-DETECT, TPAIR and Corsi-blocks in the Full Group of Participants. Participants who performed better in the TP-DETECT task exhibited higher scores on explicit auditory memory (TP-COMP), visual sequential memory (CORSI), and engagement (TPAIR).

A regression analysis was conducted on the full group of younger and older participants with TP-DETECT performance as the predicted variable, and performance on the other tasks (SiN, TP-COMP, CORSI, STEP, TPAIR, binaural/headphones task) and age as predictors. The results of the regression indicated that the model was a significant predictor of TP-DETECT performance, *F*(7, 267) = 21.522, *p* < .001, with 34.4% of the variance in the data (adjusted R^2^) explained by the predictor variables. TP-COMP (*p* < .001; standardised β = 0.211), TPAIR (*p* < .001; standardised β = 0.394) and CORSI (*p* = .009; standardised β = 0.151) added significantly to the model, confirming that these variables independently explain variance in TP-DETECT (see also Figure 6). Notably, age (*p* = .647) and the binaural/headphones test (*p* = .215) did not explain TP-DETECT variance. The lack of correlation with age here, despite the presence of an age effect in the between-group analysis (Figure 5G) might be due to shared variability between age and Corsi-blocks.

The results thus indicate that the implicit aSM capacity that supports pattern detection is positively predicted by other (explicit auditory; explicit visual) memory tasks as well as by tasks that quantify engagement and attention.

## Discussion

This study aimed to understand whether aSM capacity (as quantified primarily with TP-DETECT) is predictive of SiN reception. A related aSM task but incorporating deliberate intent to memorise (TP-COMP) was also included. We hypothesised that the link between SiN performance and aSM might be particularly salient in older listeners due to the growing individual variability in both memory capacity and SiN reception abilities in this population. To focus on the low-level sensory processes that support the extraction and maintenance of dynamically unfolding auditory information, we chose SiN and aSM tasks that minimally rely on semantic, reasoning, and executive abilities. Our results, obtained from a large cohort of online participants, showed that although both SiN perception and (to a lesser extent) aSM deteriorate with age, the two do not share variability. This finding helps constrain the search for the perceptual and cognitive factors that explain individual variability in SiN performance.

### No Shared Variability Between SiN Perception and Implicit or Explicit Sensory Memory

To specifically target sensory contributions to SiN, we used a speech perception task that minimally depends on executive processing. The test employs a closed set of keywords – colours and numbers – and a simple response procedure (see Figure 3). To measure aSM, we used two tasks, based on rapid, arbitrary tone patterns, that minimise reliance on semantic information and rehearsal. The key task tapped automatic mnemonic mechanisms supporting the detection of auditory patterns (TP-DETECT). The patterns are too rapid to allow for deliberate tracking – they pop out automatically – but the fidelity of detection depends on the properties of a sensory memory store in which current and recently encountered sensory information is processed (Harrison et al., 2020; Kaernbach, 2004; McDermott et al., 2011). To determine that a regular pattern has emerged, the auditory system must maintain the recently encountered information in some form of memory store, compare incoming information to this representation, and detect pattern repetition. This stimulus has been extensively used to study auditory short-term memory and its neural underpinnings (Barascud et al., 2016; Barczak et al., 2018; Bianco et al., 2023, 2020; Herrmann et al., 2019; Southwell & Chait, 2018). An additional task (TP-COMP), using similar stimuli, required active memory maintenance. Listeners were instructed to remember a random tone sequence over a short duration (delayed match to sample). A control visual task, Corsi-blocks, in which participants were required to actively memorise and reproduce a spatial visual sequence was also used to understand whether any memory deficits also extend to explicit visual sequence memory.

Despite having sufficient statistical power to detect low-to-moderate effect sizes, the results indicated that aSM, as tested with TP-DETECT and TP-COMP, does not predict SiN performance. This was confirmed when tested in the full group of young and older participants, and also when focusing on the older group – where we expected a potentially enhanced effect due to increased age-related variability in SiN and memory performance (Bopp & Verhaeghen, 2005; Cooper et al., 2006; Dhrruvakumar & Yathiraj, 2021; Fogerty et al., 2016; Sur & Golob, 2020). Though much attention has focused on age-related deficits in episodic and WM (Akeroyd, 2008; Cansino, 2009; Füllgrabe & Rosen, 2016), evidence is increasingly demonstrating impairment also in tasks that draw on sensory memory (Bellis et al., 2000; Bopp & Verhaeghen, 2005; Cooper et al., 2006; Dhrruvakumar & Yathiraj, 2021; Fogerty et al., 2016; Lu et al., 2005; Rimmele et al., 2012; Ruzzoli et al., 2012; Sur & Golob, 2020; Trainor & Trehub, 1989). For instance, the mismatch negativity (MMN), a pre-attentive brain response that reflects an incongruity between information stored in aSM and new input (Garrido et al., 2009; Heilbron & Chait, 2018; Näätänen et al., 2007), exhibits reduced amplitude and longer latencies in older compared to younger adults, indicating impaired aSM (Cheng et al., 2013; Cooper et al., 2006; Pekkonen et al., 1996).

Here, as expected, we observed a reduction in SiN reception in older listeners and also confirmed an age-related decline in Corsi-blocks performance (Beigneux et al., 2007; Fournet et al., 2012). We additionally revealed a potential deficit in aSM (TP-DETECT) in older listeners, though this effect was much less pronounced than that seen for SiN and Corsi-blocks (see also Figure 5). Critically, SiN perception did not share variability with these tasks.

Using stimuli similar to those at the basis of the TP-DETECT task employed here, Herrmann et al. (2019) (see also Al Jaja et al., 2020; Herrmann et al., 2022) recently demonstrated reduced auditory pattern-evoked brain responses in older listeners, relative to a young control cohort. This was interpreted as reflecting impaired age-related sensory memory and hypothesised to relate to other deficits exhibited by older listeners, including reduced SiN performance. The present results demonstrate that this is not necessarily the case, though, of course, a potential correlation between a neural measure of aSM as captured in Herrmann et al. (2022) and SiN reception is not excluded by our behavioural findings.

Finally, the failure to observe a link between SiN and aSM as measured with a pure-tone-based paradigm may be linked to some tentative evidence of two separate mnemonic subsystems for verbal material and tonal material (e.g., melodies, tone patterns) (Caclin & Tillmann, 2018). Accordingly, patients with different brain lesions exhibit double dissociation of short-term memory for tone versus syllable sequences (Hirel et al., 2017). Moreover, impaired short-term memory for non-verbal but not verbal sounds is observed in congenital amusia (Albouy et al., 2013; Tillmann et al., 2009). Therefore, the lack of shared variability between TP-DETECT and SiN outcomes may be attributed to the involvement of different networks. Sensory-specific auditory-frontal areas are known to support tone-pattern processing (Barascud et al., 2016; Herrmann et al., 2022), while cognitive frontoparietal regions are involved in memory and attention during verbal (Majerus et al., 2006) and SiN tasks (Wong et al., 2009). However, it is worth mentioning that SiN performance in normal hearing listeners has been previously linked with the processing of artificial tonal stimuli in figure-ground segregation tasks (Holmes & Griffiths, 2019). Future studies should further explore the potential extent of shared mnemonic or attentional resources during the processing of verbal and tonal material.

### SiN Performance is Predicted by Dichotic Pitch Perception and age

Analyses combining the full participant pool, or focusing on the older participants only, revealed that, from among the set of tasks used here, SiN performance was only predicted by the binaural/headphones test and participant age. Notably, this relationship was observed despite the absence of a binaural component in the SiN task itself. The binaural/headphones test (Milne et al., 2020) was included here as a standard component of our online testing protocol to screen for headphones use. This test was chosen because it is based on a salient pitch percept (the HP, Akeroyd, 2008; Chait et al., 2006; Cramer & Huggins, 1958) that is perceivable by most listeners including those with hearing loss (Akeroyd et al., 2001; Sanchez-Lopez et al., 2020; Santurette & Dau, 2012; though we note that the hearing-impaired participants tested on HP e.g., in Santurette & Dau, 2012 were not controlled for age).

We have previously used the headphones screen with predominantly young listeners (Bianco et al., 2021; Milne et al., 2020). A failure rate of around 20% is usually observed in this work, and hypothesised to result from faulty equipment (e.g., headphones not used, or the presence of bleed between L and R channels), a particularly noisy environment, or lack of engagement. Indeed, here failure on the binaural/headphones test among the young group (20% fail rates) was predicted by environmental noise, and task engagement as quantified with the TPAIR attention task. That said, it is also possible that a proportion of young listeners failed because of impaired binaural processing (Füllgrabe & Moore, 2018).

The older cohort exhibited substantially lower pass rates (40% fail rates) and as opposed to the younger cohort the binaural/headphones test was predicted only by SiN performance. This might reflect potentially impaired binaural processing in the older population which can be of a peripheral nature or of a more central origin. There exists evidence to indicate that ageing and sensorineural hearing loss independently affect binaural processing (Füllgrabe & Moore, 2018; King et al., 2014). We therefore used the outcome of the binaural/headphones test as a proxy for binaural processing ability in the reported regression analyses. Whilst it is important to stress that the binaural/headphones task was not originally designed to specifically assess binaural processing ability, the finding that performance correlated with a non-dichotic SiN task is interesting and links with previous observations (Sanchez Lopez et al., 2018).

A previous study (Sanchez Lopez et al., 2018) investigated a data-driven approach for profiling individuals based on a range of auditory ‘supra threshold’ tasks (see also Sanchez-Lopez et al., 2020; Wu et al., 2020). They classified listeners into four auditory profiles (A, B, C, and D), based on perceptual performance along two independent computationally derived dimensions. The first dimension was associated with high-frequency hearing loss and reduced speech intelligibility; the second was associated with low-frequency hearing loss and impaired loudness perception. Strikingly, results showed that only individuals exhibiting clear deficits in both dimensions (‘profile C’), that is, those with the most severe hearing loss, displayed reduced binaural pitch perception. This indicates that our online sample might contain such individuals (despite self-reported normal hearing), and that, more broadly, this profile might characterise about 20% of older listeners when sampled randomly. Therefore, it may be valuable to routinely incorporate a measure of binaural processing when working with older listeners, even if the tasks of interest are not inherently binaural in nature. Notably, it was observed that individuals who exhibited poorer performance on the SiN task and the binaural/headphones test did not show signs of memory impairment that would affect their performance on the TP-DETECT and TP-COMP tasks. The regression analyses indicated that sensitivity to the binaural pitch did not predict performance on TP-DETECT and TP-COMP, in contrast to its predictive value for SiN performance. This indicates that the brain ageing processes that underlie impaired SiN and binaural processing are not necessarily also associated with reduced aSM.

### Mildly Impaired Auditory Sensory Memory With Ageing

We used auditory implicit and deliberate sensory memory tasks to quantify listeners’ ability to maintain sequential events in memory. Unlike previously used probes of auditory memory that relied on listeners’ ability to explicitly recall the order of sequentially presented auditory information (e.g., Dhrruvakumar & Yathiraj, 2021; Fogerty et al., 2016), the present tasks were based on rapidly presented (20 Hz) sequences of tone pips and focused on listeners’ ability to implicitly represent the sequence as a whole. Performance on all tasks yielded substantial individual variability (Figure 5) with participant performance spanning the full range between chance and ceiling.

The TP-DETECT task is arguably a direct measure of aSM because it is implicit and does not involve an active retention component. It is tempting to hypothesise that the memory mechanisms that support the automatic detection of structure in rapidly unfolding sequences would also support other listening tasks, such as SiN reception (Herrmann et al., 2022).

Despite the widely reported decrease in aSM with ageing (Bellis et al., 2000; Bopp & Verhaeghen, 2005; Cooper et al., 2006; Dhrruvakumar & Yathiraj, 2021; Fogerty et al., 2016; Lu et al., 2005; Rimmele et al., 2012; Ruzzoli et al., 2012; Sur & Golob, 2020; Trainor & Trehub, 1989) we observed only relatively mild effects in the present study (Figure 5G). Performance on the delayed-match-to-sample task (TP-COMP) did not differ significantly between age groups. Though evidence suggested a difference between age groups for the implicit memory task (TP-DETECT), the effect size was much smaller than those observed for SiN and Corsi-blocks. This indicates that aSM, at least when quantified with d’, is relatively preserved with ageing.

These findings stand in contrast to the quite pronounced EEG-based effects observed in Herrmann et al. (2019) who, using signals akin to those used for TP-DETECT here, showed substantially reduced auditory pattern-evoked sustained brain responses in older, relative to younger, listeners (see also Al Jaja et al., 2020; Herrmann et al., 2022). This discrepancy might suggest that the reduced responses observed in those studies do not directly underlie memory performance as measured behaviourally. Alternatively, finer-grained measures of sensitivity (e.g., measuring reaction time to patterns of increasing length) may be more informative for quantifying deficits in aSM. We chose to focus on sensitivity (as quantified with d’) here because it is easy to measure online and because participants exhibited a sufficiently large, and thus potentially informative, individual variability in performance.

### Links Between Auditory Implicit and Explicit Sensory Memory and Visual Memory

Across older and younger listeners, performance on the TP-DETECT task, our key measure of automatic aSM, was positively predicted by the TP-COMP task (explicit aSM) and the Corsi blocks task (explicit visual short-term memory). The TP-DETECT task requires information to be held in memory to detect a pattern repetition, but due to the rapid nature of the stimuli (a presentation rate of 20Hz here), the process of detection is largely implicit (the pattern ‘pops out’ perceptually). Therefore, the shared variability between the memory tasks may reflect the contribution of a task-independent, and modality-independent memory component. This finding is consistent with demonstrations that auditory and visual short-term memory employ similar fundamental information processing steps (Visscher et al., 2007) and therefore might be constrained by similar, individual-linked capacity limitations.

Furthermore, a portion of the variance in TP-DETECT performance was also explained by the control TPAIR task. Although this may partly reflect the contribution of frequency representation to pattern detection performance, it is more likely attributed to attentive listening and task engagement. This interpretation is supported by the nature of the TPAIR task, which is relatively easy (noticing a frequency difference of 12% between tones in a pair) but repetitive, and demanding sustained focused attention. Performance lapses are more likely due to inattention rather than genuine insensitivity. It is worth noting that, contrary to expectations associated with a task solely reflecting frequency sensitivity (Moore & Peters, 1992), performance on TPAIR did not correlate with age (Spearman's rho = 0.0: see also Figure 5).

## Conclusion

There is currently significant interest in investigating the relationship between auditory WM and speech processing. In this study, we aimed to specifically examine the aSM component and explore the association of implicit and deliberate aSM with SiN performance. Despite considerable variability in SiN and auditory memory task performance across a large cohort of younger and older participants, our findings indicate that SiN performance was not predicted by aSM alone. This suggests that the previously observed links between auditory WM and SiN performance are not solely reliant on the maintenance of acoustic information in memory, but rather involve executive and other supportive mechanisms.

It is essential to acknowledge the limitations associated with conducting an online study. To gather a large sample size, we had to relinquish control over participants’ equipment and environment, and our knowledge of their audiological profiles was limited. Although we took steps to mitigate some of these limitations (e.g., excluding participants who reported particularly noisy environments, incorporating loudness adjustment before each task, and using the binaural/headphones test as a predictor in all analyses), conducting more detailed in-lab studies could provide further insights into how a listener's specific audiological and cognitive profile influences the observed effects.
